# Advantages of digital twin technology in orthopedic trauma Surgery – Exploring different clinical use cases

**DOI:** 10.1038/s41598-025-04792-w

**Published:** 2025-06-06

**Authors:** Annchristin Andres, Michael Roland, Kerstin Wickert, Stefan Diebels, Johannes Stöckl, Stefan Herrmann, Frank Reinauer, Ralf Leibinger, Atanas Pavlov, Lutz Schuppener, Daniel Schäfer, Tina Histing, Benedikt J. Braun

**Affiliations:** 1 Saarland University, Applied Mechanics, Saarbrücken, Germany; 2 csi entwicklungstechnik GmbH, Neckarsulm, Germany; 3 KLS Martin SE & Co. KG, Tuttlingen, Germany; 4 IL Innovationslabor GmbH, Berlin, Germany; 5 Faculty of Medicine, University Hospital Tuebingen on Behalf of the Eberhard-Karls- University Tuebingen, BG Hospital Tuebingen, Tuebingen, Germany

**Keywords:** Digital twin, Virtual reconstruction, Patient-specific model generation, Individualized Biomechanical simulation, Fracture healing, Non-unions, Surgical treatment., Fracture repair, Computational models

## Abstract

Digital twin technology offers significant benefits for addressing fracture non-unions in orthopedic trauma surgery, particularly in cases requiring revision surgery. In this study, we developed a clinically applicable digital twin workflow that integrates patient-specific imaging, motion capture, musculoskeletal modeling, and finite element simulation. We applied this workflow to five real patient cases involving different anatomical sites and treatment strategies, including implant modification, augmentative fixation, and corrective osteotomy. Each case was virtually reconstructed to evaluate mechanical conditions before and after revision surgery. The results demonstrated that digital twins can predict improvements in implant stress distribution and fracture strain states, offering valuable insights for optimizing surgical decisions. This work highlights the feasibility of digital twins and their clinical value in supporting individualized revision strategies, laying the foundation for their broader use in trauma care. .

**Supplementary Information:**

The online version contains supplementary material available at 10.1038/s41598-025-04792-w.

## Introduction

Since its first appearance in 2003^1^, digital twin (DT) technology has rapidly evolved as a field in medicine, enabling significant advances by transferring established concepts from engineering science and industry into clinical practice^2^. By generating virtual models that mirror the underlying physical entities, DTs currently enable the simulation of patient-specific issues within pre-defined application-related frameworks, thereby predicting health outcomes and enhancing diagnosis, treatment, and preventive care.

This wide spectrum encompasses diverse applications across several medical fields. In general healthcare and personalized medicine, DTs have been used in geriatric medicine^3^, to identify optimal drug treatments^4^, and for public health management^5,6^. DTs are also applied to cancer care^7^, oncology workflows^8,9^, and in radiology^10^. Additionally, DTs are used for investigating vertebrae and vertebroplasty procedures in cancer patients^11,12^, for predicting the electrical field in cell culture experiments with human osteoblasts^13^, and for osteoporosis research, particularly in the context of denosumab therapy^14,15^.

Surgical planning applications have also emerged, particularly in oral and maxillofacial surgery. These include mandibular reconstruction using computer-aided design and computer-aided manufacturing (CAD/CAM) techniques alongside fibula flaps to create patient-specific reconstruction plates^16^, or to prevent biologically inadequate reconstructions that lead to functional and aesthetic impairments^17^, as well as cranial defect reconstructions with tailored resorbable implants^18^. DTs are also used in orthodontics, for example, in implant placement^19^ and for simulating the human mandible after joint replacement^20,21^. Despite all progress, DTs are still underrepresented in orthopedic trauma surgery.

A PubMed search at the end of 2024 combining (“digital twin” OR “virtual twin”) AND “fracture” AND (“tibia” OR “femur” OR “bone”) yielded fewer than 20 articles, several of which were unrelated to medicine. This also included review articles and a viewpoint addressing arthroscopic knee surgery^22^, virtual reality in bone trauma procedures^23^, digital technologies in orthopedics, with a focus on bone fracture^24^, and the potential of integrating digital technologies into computer-assisted surgery^25^.

Today, DTs in orthopedic trauma surgery are primarily based on 3D models of patient anatomy derived from clinical imaging to generate workflows for decision support, individualized product development, and scientific research.

The closest related work to this study is that of Aubert et al. (2021)^26^, which presented a DT approach to optimize trauma surgery and postoperative management in tibial plateau fractures. While their study was a significant step forward in demonstrating the value of simulation for treatment planning, it focused on a narrow anatomical region, did not incorporate real patient-specific motion capture data, and offered limited integration into clinical surgical workflows. This study expands upon this by applying DTs across multiple anatomical sites and revision strategies, incorporating dynamic musculoskeletal simulations and real-world clinical imaging, and aiming for a more holistic decision-support system tailored for orthopedic trauma care.

If the literature search were extended to include terms such as “simulation” OR “finite element analysis” instead of DT, many more studies and articles would be found. Here, the review by Ghiasi and colleagues^27^ provides a good first overview.

Although personalization and individualization of therapy through DTs are among the most promising trends in digital healthcare, there is still no consensus on standardizing or designing such systems for clinical use^28^. Human DTs are often loosely defined as “computer models of humans tailored to any patient,” without specifying the necessary clinical, mechanical, or biological fidelity^29^. In orthopedic trauma, these challenges are compounded by the complexity of bone healing mechanics, variability in patient anatomy, and the practical limitations of clinical imaging. As Killen et al.^30^ highlight, even well-established musculoskeletal modeling tools remain underutilized in practice due to barriers such as integration at the point of care, the need for specialized expertise, and limited clinical familiarity with simulation-based planning. Our work addresses some of these challenges by proposing a structured, patient-specific DT pipeline and demonstrating its feasibility in realistic clinical scenarios.

The goal of this study was to enhance treatment planning for non-union patients by utilizing a digital twin-based process chain. This approach integrates individualized computational models with patient-specific motion capture and clinical imaging data to create a data-driven DT. The resulting twin captures physical, biological, and treatment history data, enabling more informed and reliable surgical decision-making for revision procedures. The aim is to demonstrate how DTs can be utilized specifically in evaluating potential revision strategies and how they could provide decision support in the future.

Fracture revisions present a particularly complex problem for DT applications due to a combination of biological variability, incomplete healing histories, altered implant environments, and the often uncertain mechanical integrity of previous treatments. Unlike primary fractures, revision surgeries must address failed healing and suboptimal loading conditions, often without access to the original surgical data or implant CAD models. Furthermore, there is limited guidance on how to integrate patient-specific motion data, simulate realistic mechanical environments, or evaluate multiple revision strategies in silico. In this context, our work aims to develop and demonstrate a clinically oriented DT workflow capable of reconstructing both initial and revised treatment scenarios using real patient data, musculoskeletal modeling, and finite element analysis. By doing so, we seek to provide a reproducible, biomechanically validated framework for decision support in orthopedic fracture revision surgery. This study represents a step toward realizing predictive, patient-specific DT for preoperative planning in complex trauma care.

## Results

Five cases were virtually processed using DT technology to evaluate different treatment strategies and loading scenarios, focusing on mechanical fracture healing conditions. The following sections present the main results, and the supplementary material provides more detailed information about the patients and the monitoring process. The primary objective in every case was to recreate the initial treatment situation based on current computed tomography (CT) data, analyzing the treatment’s mechanical stability and the fracture’s strain state. The revision strategy was then virtually modeled and, analogous to the initial treatment, mechanically analyzed and evaluated regarding improving healing chances.

### Use case 1 - Variation of the thickness of an intramedullary nail

**Fig. 1:**
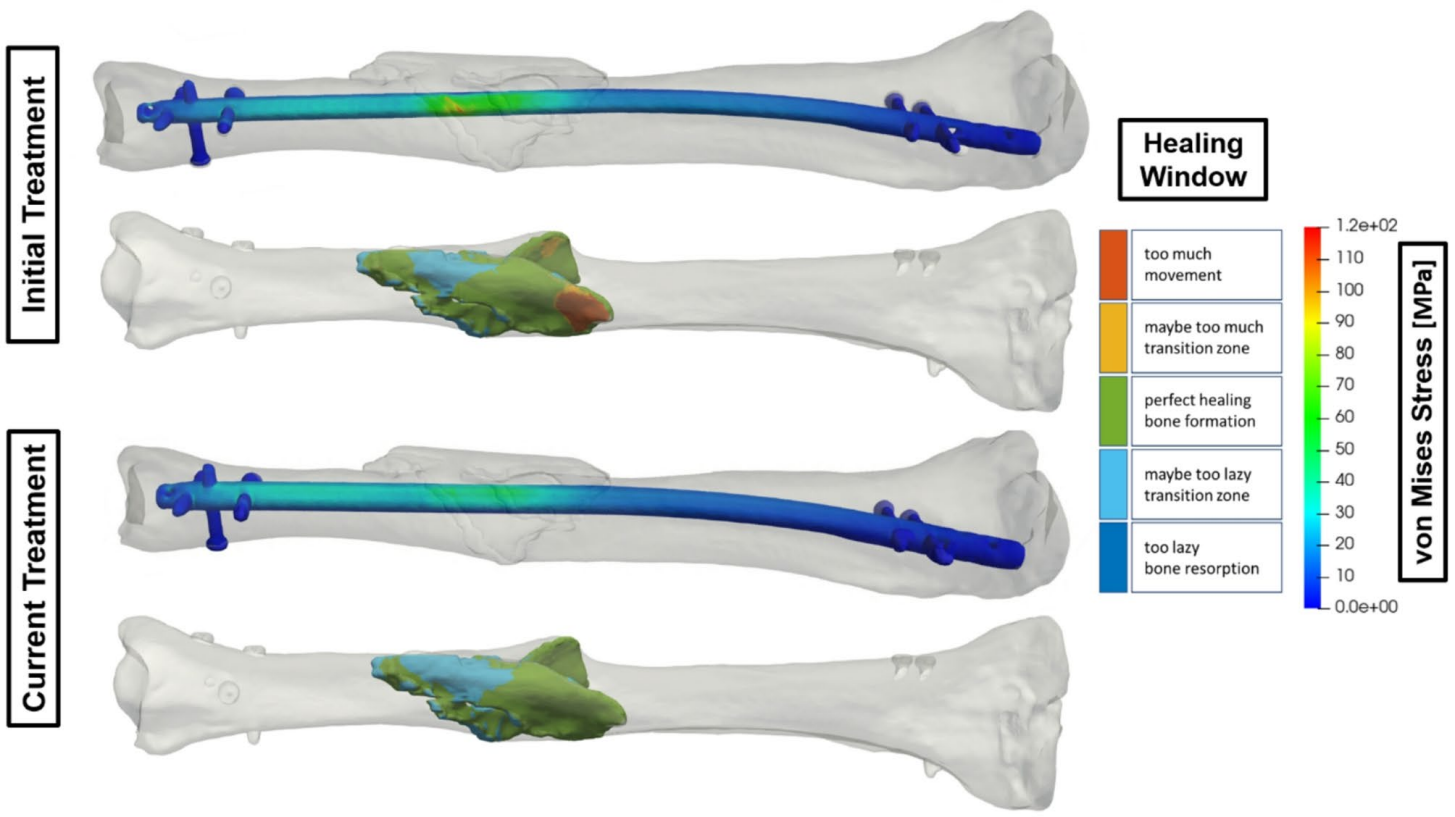
Comparison of the strain state-based healing window in the fracture and the von Mises stress distribution for a diameter change of an intramedullary nail.

Non-union of the tibial shaft occurs when a fracture fails to heal properly. One treatment strategy involves changing the nail size to stabilize the bone, as shown in Fig. 1. Here, the surgeon replaced an 8 mm nail (initial treatment) with an 11 mm nail (current treatment) after reaming, which removes bone material to accommodate the larger nail. The treatment plan recommended pain-adapted, full weight-bearing without restrictions to promote healing. In the DT for this use case, the diameter of the original nail was virtually modified without altering its position. Biomechanical analysis revealed a significant reduction in implant von Mises stress, exceeding 50%, achieved by simply thickening the nail by 3 mm in diameter. This modification alleviated the fracture and highlighted the importance of structural changes in optimizing mechanical outcomes. The improvement was evident from the healing window, where the initial treatment had 7% of the volume of the fracture in the range of too much movement.

## Use case 2 – Augmentative plate for a humeral fracture

**Fig. 2:**
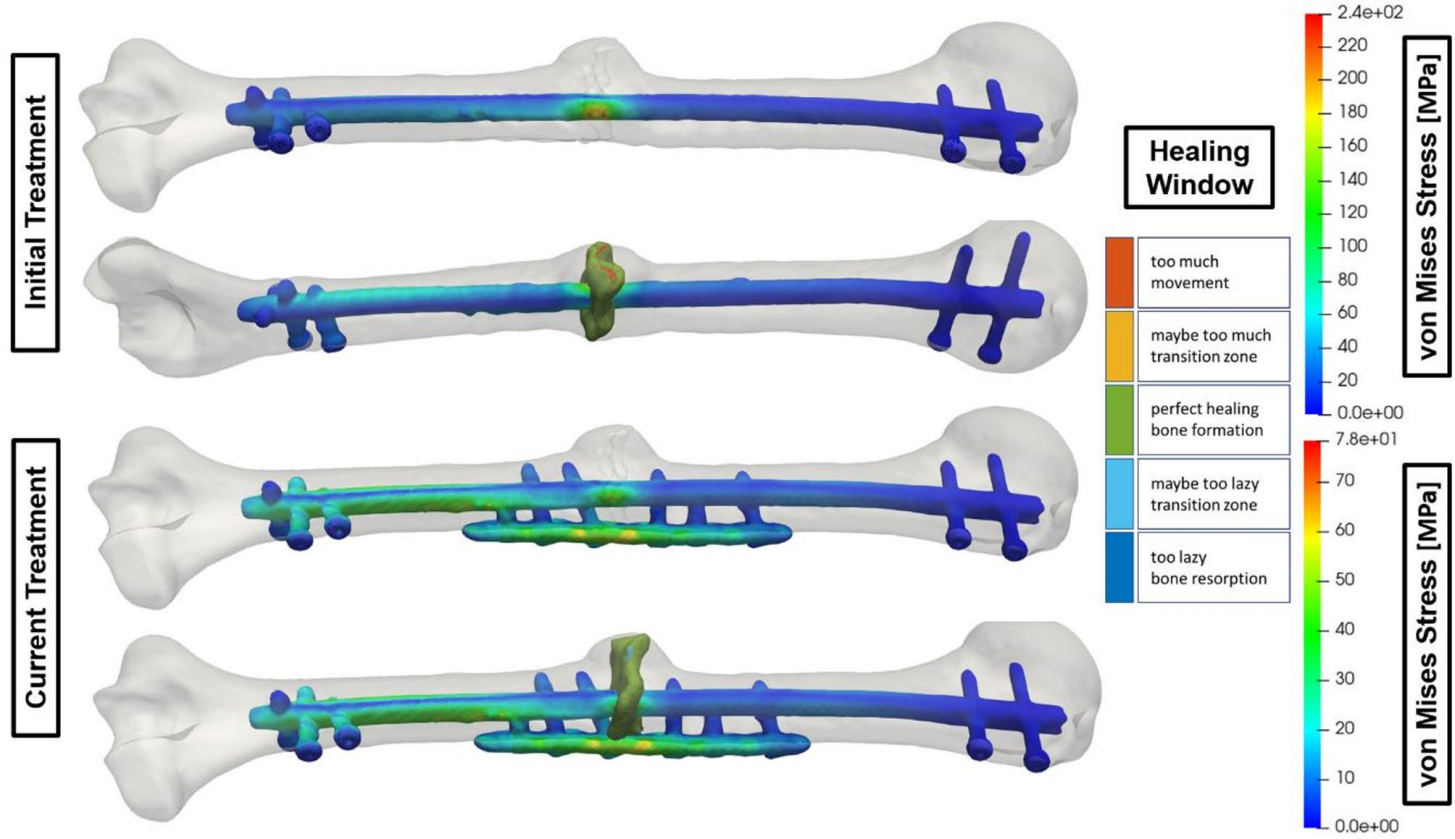
Comparison of the fracture’s strain state-based healing window and the treatment’s von Mises stress distribution with and without an adaptive humerus plate.

Surgeons have proposed augmentative plating as an alternative to nailing, nail removal, and plate revision. In the following use cases, 2 and 3, the fractures were stabilized using an additional plate, improving the fracture situation. This technique is effective for non-union revision surgeries in both the lower and upper extremities. The results show a significant reduction in the von Mises stress in the central area of the implant examined using an augmentative plate combined with an intramedullary nail for a right humeral fracture (Fig. 2). In the initial treatment, the maximum von Mises stress of the implant reached 240 MPa, indicating a high mechanical loading. However, adding the augmentative plate reduced the stress maximum to 78 MPa, marking a significant improvement. This reduction by a factor of approximately 3 enhances the material’s structural integrity and demonstrates the effectiveness of the treatment in reducing stress concentrations and improving mechanical performance. We used standard titanium alloy implants, which have an ultimate tensile strength slightly above the 860 to 925 MPa range, to obtain approval (e.g., DIN EN ISO 5832-3).

The 240 MPa, representing roughly 25% of the maximum load, explains why the implant remained intact, although prolonged loading could lead to fatigue and failure. While standard titanium alloys (e.g., Ti-6 Al-4 V) may have ultimate strengths above 860 MPa, their yield strengths often range between 240 and 550 MPa, depending on alloy and heat treatment, making the observed stress level clinically relevant. Therefore, the 78 MPa achieved in the new treatment is the better option. This also aligns with the healing window, reducing the elements in the range of too much movement to 0%.

## Use case 3 – Augmentative plate for a femoral fracture

**Fig. 3:**
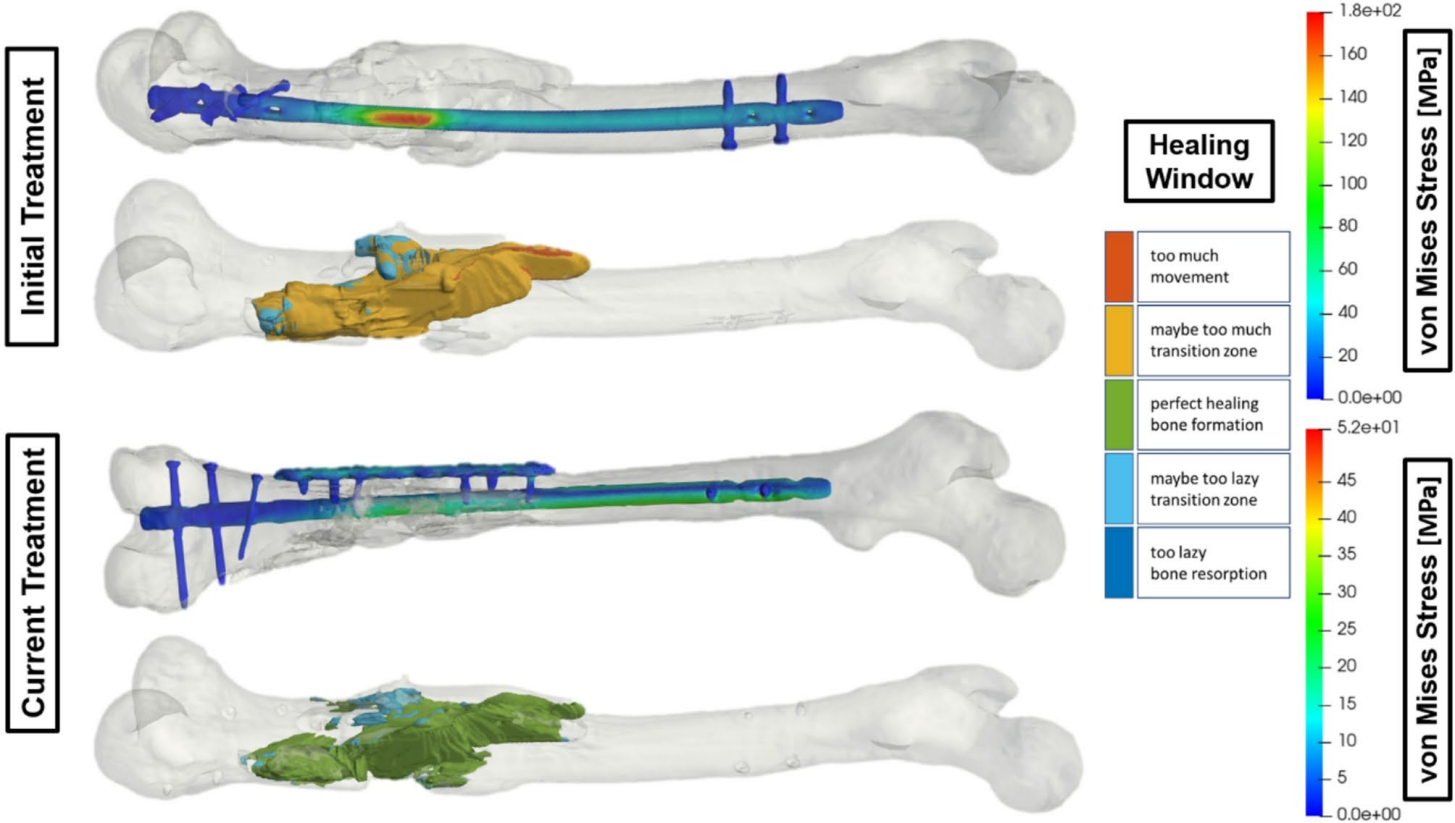
Comparison of the strain state-based healing window of the fracture and the von Mises stress distribution of the treatment with and without an adaptive femur plate.

Figure 3 shows an augmentative plate for a femur fracture treated with an intramedullary nail. In the initial treatment, the von Mises stress maximum of 160 MPa on the nail is located at the center of the fracture, leading to instability and excessive fragment movement. Adding the augmentative plate significantly decreased the stress maximum to 30 MPa. The DT process chain includes virtually removing the additive plate and screws to recreate the original situation, address the instability from the initial treatment, and explore ways to improve the mechanical conditions for fracture healing. The current treatment, which incorporates a reinforcing plate, adds substantial strength and effectively reduces stress. As a result, fracture fragment movement reduces to the bone healing window without entering the transitional zone of bone resorption.

## Use case 4 – Additional screw for a tibia fracture

**Fig. 4:**
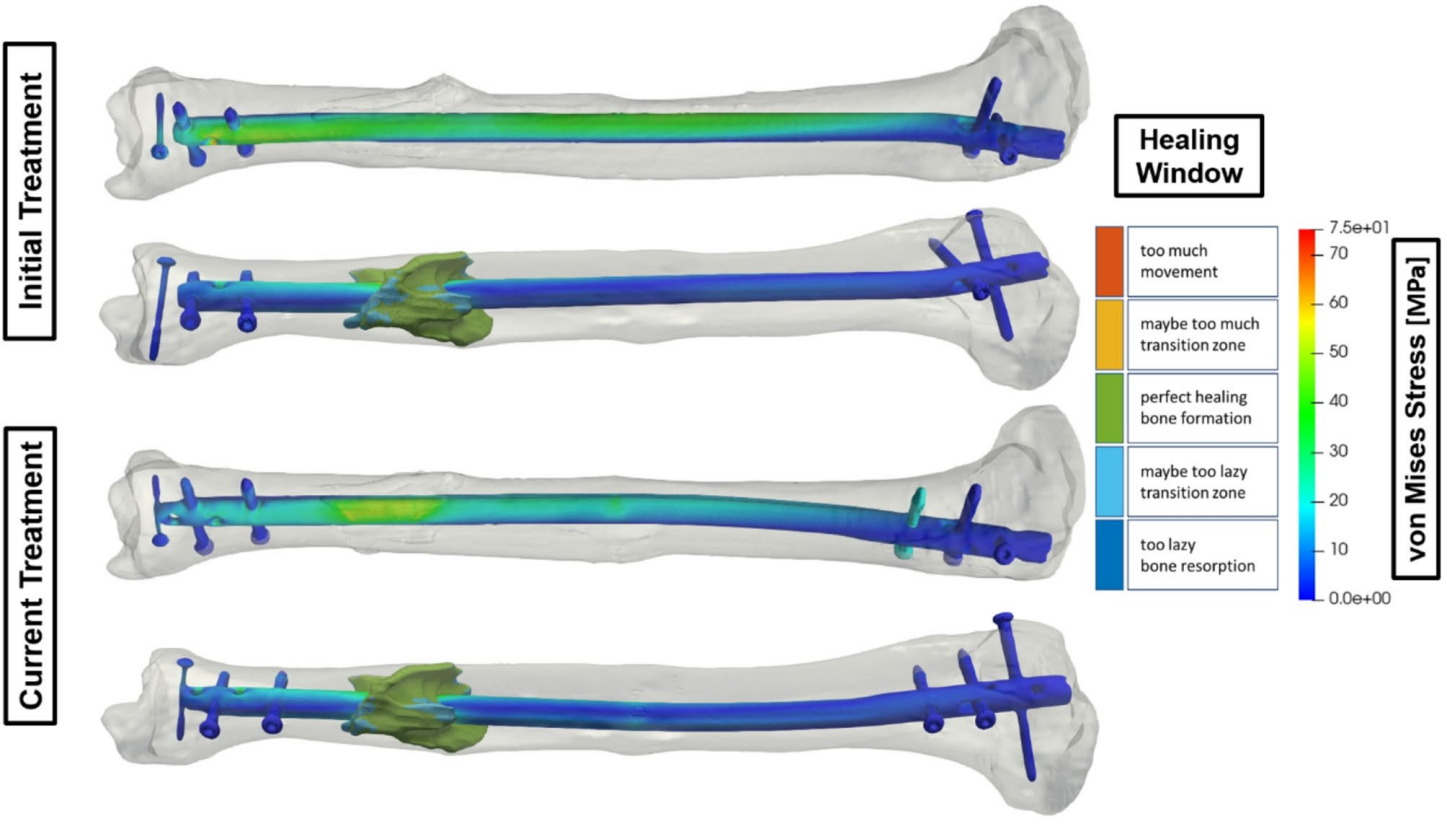
Comparison of the strain state-based healing window of the fracture and the von Mises stress distribution of the treatment with and without an additional screw.

Integrating additional screws, so-called strain reduction screws, in tibia fractures treated with intramedullary nails offers a refined approach to fracture management, as shown in Fig. 4. By enhancing the primary stabilization provided by the intramedullary nail, the supplementary screw improves the overall construct stability and provides targeted reinforcement at critical fracture sites. This strategy addresses various fracture patterns and patient-specific biomechanics, thereby contributing to faster healing and reduced complications. The torsion correction achieved by the additional screw increases the percentage of elements in the optimal healing window from 78 to 86%.

## Use case 5 – Wrist movement after implant individualization

Use Case 5 represents a corrective osteotomy rather than a classical non-union case. In this scenario, the patient exhibited a malaligned wrist following prior treatment, impairing joint kinematics. The DT simulated pre- and postoperative wrist motion, aiding in the realignment strategy to restore normal biomechanics. Before the intervention, clinicians assessed the patient’s range of motion using the described motion-capturing process. This preoperative analysis revealed restricted radial and ulnar deviation angles compared to the clinically established target ranges of 20–30 degrees for radial deviation and 30–40 degrees for ulnar deviation. The calculation determined the deviation angles as the angle between the anatomical axis of the radius and the vector representing the combined motion of the proximal carpal unit, defined as a rigid body including the scaphoid, lunate, and triquetrum. The virtual representation consisted of deformable and rigid components to simulate these conditions. The model treated the radius, ulna, and implant as deformable, while representing the scaphoid, lunate, and triquetrum as a single rigid unit.

**Fig. 5:**
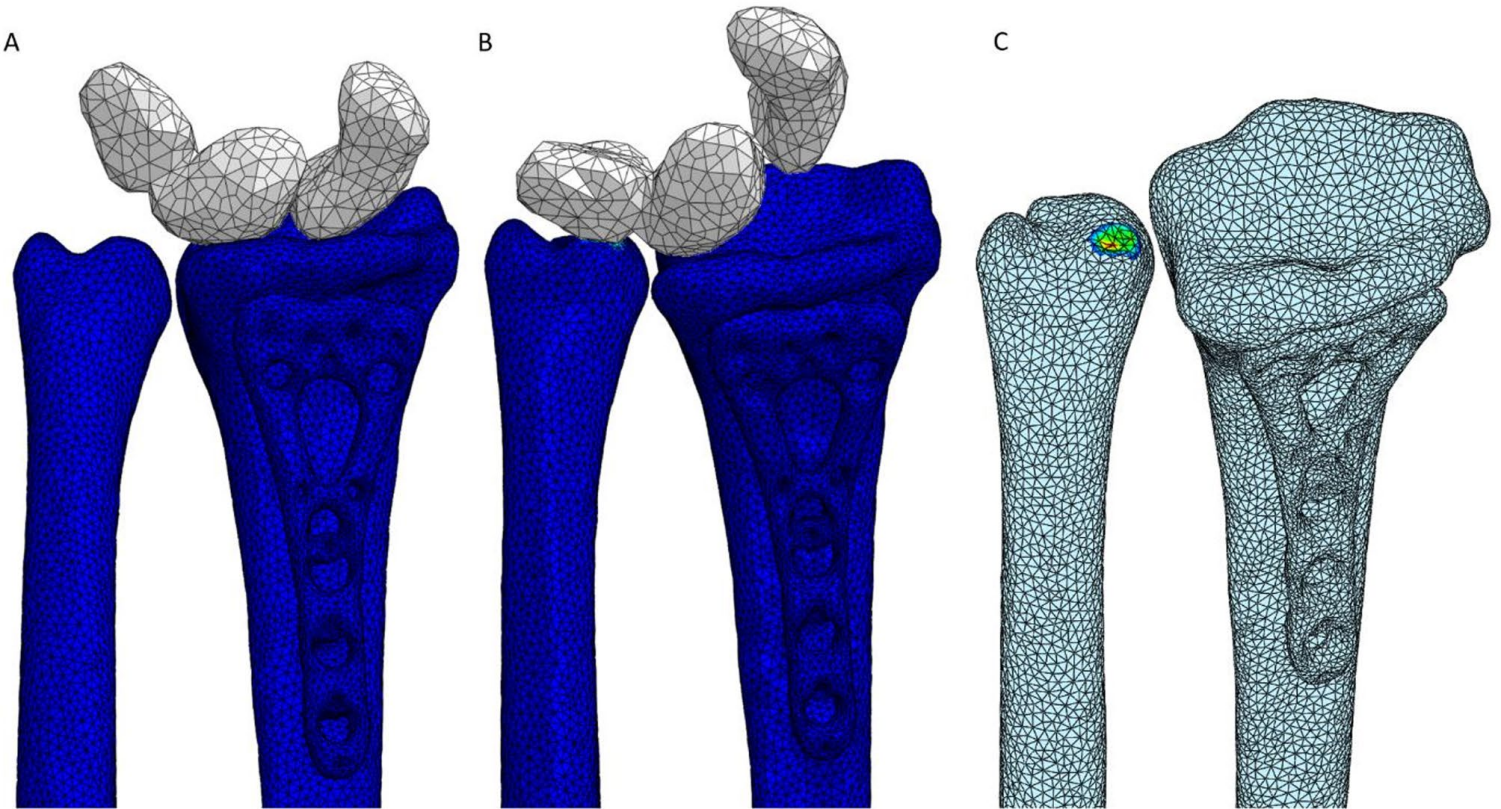
Preoperative case, A: 0-degree ulnar deviation, B: 40-degree ulnar deviation, C: contact during the 40-degree ulnar deviation with fade-out rigid body part for better visualization.

**Fig. 6:**
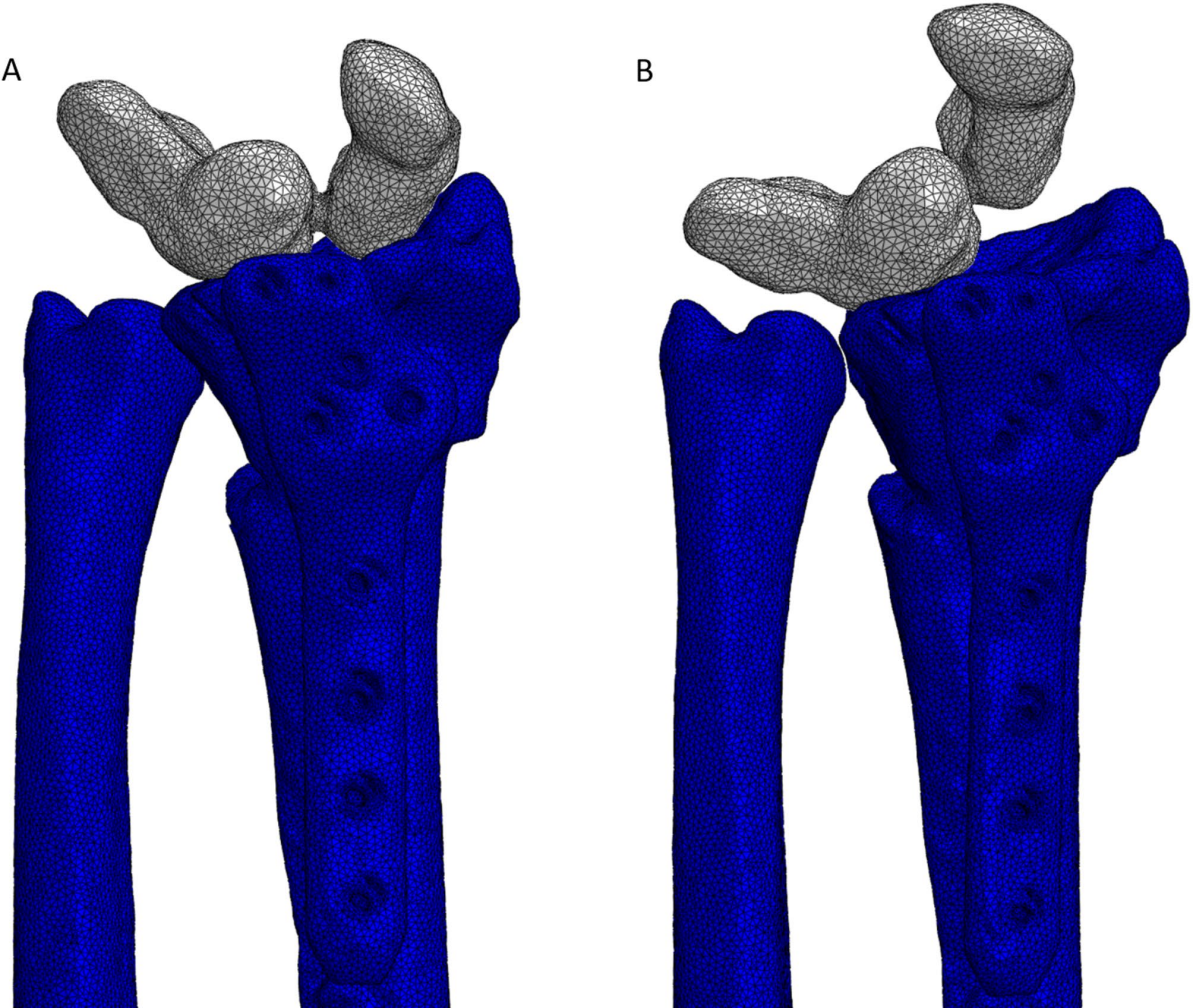
Postoperative case, A: 0-degree ulnar deviation, B: 40-degree ulnar deviation.

Preoperatively, malalignment between the radius and ulna hindered attempts to achieve 40 degrees of ulnar deviation. This misalignment resulted in direct contact, seen in Fig. 5, between the carpal unit and the radius at higher deviation angles, thereby limiting the joint’s functional range and preventing full rotational movement. Following the reconstructive surgery shown in Fig. 6, the realignment of the radius and optimization of implant positioning enabled the patient’s wrist to move without impingement throughout the specified range. The procedure resulted in no structural interference when the wrist was positioned at 0 degrees. More importantly, when attempting 40 degrees of ulnar deviation, the configuration allowed full movement without any contact between the carpal bones and the ulna, indicating that the corrective measures had successfully restored normal joint mechanics. This outcome highlights the effectiveness of surgical intervention in improving joint mobility and restoring the functional range of motion in the patient’s wrist. A process that can be largely tested and visualized in advance using DT technology.

## Discussion

Integrating DT concepts into treatment strategies enables a dynamic and evolving representation of the patient’s bone-implant system. The DT evolves in tandem with the patient’s condition and treatment progress, continuously incorporating clinical imaging data, patient metadata, and biomechanical simulations. Mechanical simulations embedded within this DT framework offer a range of benefits, particularly in analyzing the micro-mechanics of fracture gaps and the associated healing process. They enhance our understanding of fracture mechanics, enabling researchers and clinicians to examine complex interactions between patient-specific anatomy, implants, and rehabilitation protocols with unprecedented detail. This holistic, data-driven approach enhances the accuracy of healing predictions and supports tailoring treatment strategies to individual patient needs. In this study, we analyzed five distinct clinical cases using DT technology to show different strategies for non-union treatment. Each case demonstrated a clear biomechanical benefit from the simulated intervention, ranging from stress reduction in implants (Cases 2–3), improved strain states in fractures (Cases 1, 4), to functional restoration of joint mobility (Case 5). These results underscore the adaptability of DTs to individual treatment challenges and patient-specific variations. For instance, while nail diameter optimization in tibial fractures (Case 1) improved load transfer, the augmentative plating techniques (Cases 2–3) enhanced construct stability, and screw augmentation (Case 4) contributed to refined strain control. Notably, wrist mobility improvement in Case 5 exemplifies how DTs can simulate functional outcomes preoperatively.

Similarities across cases include the ability to evaluate mechanical performance under real patient-specific boundary conditions. Differences arise from anatomical locations, implant configurations, and motion dynamics. Our approach contrasts with Aubert et al.^26^, which focused primarily on tibial plateau fractures and early-stage DT concepts; in contrast, our work applies a mature DT pipeline incorporating musculoskeletal dynamics and postoperative motion analysis.

While the first three cases demonstrate improvements in mechanical stability through implant reinforcement, such as increasing nail diameter or adding plates, these choices, though mechanically beneficial, may not universally translate into better clinical outcomes. Clinically, increased implant stiffness can lead to stress shielding, delayed healing, or unnecessary surgical burden. Therefore, stress reduction alone is insufficient for optimal treatment selection.

Within the DT environment, clinicians can efficiently test various implant configurations and rehabilitation scenarios, making informed decisions for specific fracture types and patient profiles in a safe and controlled environment. The ability to rapidly run these simulations reduces costs, improves turnaround time, and enhances the personalization of care, making DT-based simulations a compelling option for advancing orthopedic trauma surgery treatments. By modeling the fracture gap and simulating the biomechanical environment over time, DTs facilitate a more accurate estimation of the healing window. This helps guide interventions such as adjusting nail diameters, modifying plate constructs, or adding screws to improve stability, actions that can be informed by real-time feedback and continuous patient data integration. Similarly, by incorporating patient-specific movement data and boundary conditions, DTs shed light on the interaction between implants and native bone structure, helping determine when to intervene, how to optimize stress and strain distribution, and how to minimize complications. The adaptability of the DT to evolving clinical states underscores its potential to accurately represent the patient’s biomechanical and biological milieu. A promising future application of DT lies in in silico clinical trials, where simulations may supplement traditional clinical data and, in select scenarios, help reduce reliance on early-stage human trials, particularly in rare or high-risk conditions^7^. DT of real patients can be used to create multiple problem-specific cohorts customized to address specific medical conditions, some of which may be impossible or unethical to replicate in physical clinical trials. Consequently, conditions that remain untestable in the physical world can now be explored virtually. In the context of osteosynthesis devices (e.g., plates and screws) utilized in orthopedic trauma surgery, in silico trials with DT present a valuable opportunity for the development of novel, innovative products for specialized applications. Because these specialized products serve small patient populations and have low production volumes, the high costs of regulatory certification often make them commercially unfeasible. By leveraging DT for in silico trials, manufacturers can gather robust data on safety and efficacy without the high financial barriers associated with traditional clinical testing.

Despite the advantages offered by DTs, certain limitations persist. One key constraint is the reliance on manual segmentation processes due to the unavailability of original CAD data for implants in the scientific community. While segmented implant models can be derived from image data, the lack of direct CAD geometries hinders the precision and fidelity of implant representation. CAD-based models offer higher geometric accuracy, streamline simulations, and reduce manual modeling efforts.

Furthermore, the standard imaging protocols currently employed present several challenges. Calibration phantoms, which could ensure more reliable mapping of bone material properties, are not routinely used. As a result, bone properties must often be calibrated indirectly, which can potentially reduce the accuracy of subsequent simulations. In addition, CT scans, usually the imaging modality of choice, may have suboptimal quality and only capture the medically relevant segment of the extremity. This incomplete coverage frequently necessitates manual post-processing and extrapolation beyond the scanned region. Moreover, due to concerns about radiation exposure, the number of CT scans per patient is understandably limited. This benefits patients and their well-being, but it makes it difficult to evaluate simulations at different time points during the healing process. Continuous, longitudinal monitoring over several months to capture the healing process at fixed intervals requires multiple motion-capturing appointments, making it costly and time-consuming. These imaging and data availability constraints reduce the granularity and consistency of a DT, ultimately limiting its capacity to capture the complete patient-specific healing trajectory. A critical limitation of this approach, inherent to all patient-specific simulation-based analyses in orthopedics, is the lack of direct in vivo validation for stress and strain predictions. Unlike standard engineering applications, there is no practical or ethical way to measure internal mechanical states (e.g., interfragmentary strain, implant stresses) in live patients. As such, model predictions rely on boundary conditions derived from motion capture and musculoskeletal simulations, which are themselves estimations. Although these simulations provide clinically useful insights, they must not be interpreted as absolute truths. Instead, they should be decision-support tools that assist, but never replace, experienced surgical judgment. We therefore recommend that all results from DT-based simulations be viewed with appropriate skepticism and always considered in conjunction with clinical findings and expert interpretation. In the paper by Wickert et al.^31^ from our working group, we address the validation and verification of our simulations. Furthermore, we follow the concepts of what makes a good simulation published by Augat et al.^32,33^. Regarding a comparison with the clinical outcome, all five patients are fully healed after their revision surgery and have regained their full freedom of movement. The last follow-up appointments took place in 2022 for use case 2, in 2023 for use cases 1 and 3, and in 2024 for use cases 4 and 5.

The introduction of DTs in patient care, combined with mechanical simulations and precise biomechanical measurements, offers a patient-centric and data-rich paradigm that significantly enhances treatment outcomes. By analyzing the fracture gap in relation to the healing window through the DT, healthcare professionals gain insights that inform treatment decisions, facilitate patient involvement, and promote optimal healing trajectories. The DT’s ability to simulate individual movement and patient-specific boundary conditions reinforces its role in refining surgical strategies and rehabilitation protocols. By enabling clinicians to explore various implant scenarios and track patient progress continuously pre-emptively, the DT paradigm exemplifies a synergistic relationship between cutting-edge computational tools, patient-specific modeling, and evidence-based medical care. This integrated approach improves the quality and efficiency of patient-centered interventions.

Future applications of DTs should integrate additional decision metrics, including bone-implant stiffness mismatch, expected surgical invasiveness, risk of soft tissue disruption, healing biology, and long-term implant performance. These factors could be modeled through extensions to the current framework, such as bone remodeling predictions, patient-specific healing simulations, or surgical time estimators. Ultimately, DTs are best viewed as multi-factorial planning tools that simulate what is mechanically optimal and what is surgically appropriate for the individual patient. Longitudinal DTs could also inform rehabilitation strategies and support in silico clinical trials to complement traditional studies, particularly for rare or complex orthopedic conditions.

## Methods

**Fig. 7:**
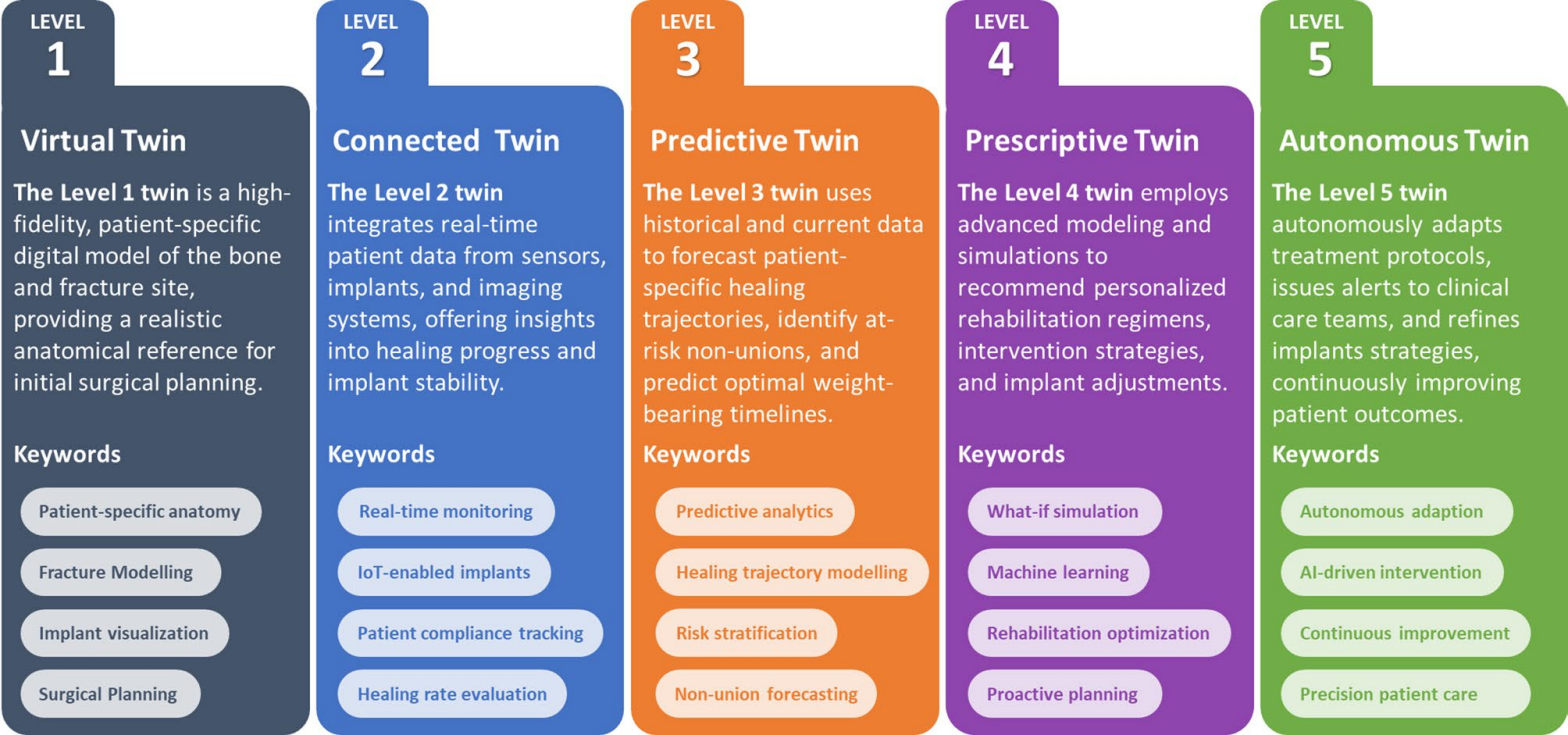
The consortium introduces different levels of digital twin maturity.

The DT concept presented in this study builds upon an innovative digital process chain^34–36^ developed and implemented by the authors and colleagues in the clinical environment at a level 1 trauma center in Germany. This concept incorporates elements from multiple levels of DT maturity, as illustrated in Fig. 7.

The DT concept used in this study integrates multiple maturity levels as defined in Fig. 7. Specifically, “Level 1 – Virtual Twin” is realized through patient-specific imaging, metadata, and CAD-based 3D models. “Level 2 – Connected Twin” is achieved by linking these models with patient-specific motion data from the musculoskeletal system. Additionally, the workflow incorporates aspects of a “Level 3 – Predictive Twin” by enabling semi-automated analysis of simulation outputs to support clinical decision-making. Future developments aim to advance toward Level 4 (Prescriptive Twin) by providing tailored real-time recommendations for patients during rehabilitation phases, and toward Level 5 (Autonomous Twin) by integrating artificial intelligence into the DT for continuous, adaptive treatment optimization.

## Patient metadata

Comprehensive metadata is collected upon the patient’s consultation at the clinic. This includes anthropometric information such as age, sex, body height, body weight, and body mass index, along with the AO classification of the fracture^37^. The patient’s treatment history is documented, including previous surgeries, revision procedures, and prior rehabilitation efforts. Based on this information, the revision surgery is planned, explicit treatment goals are defined (e.g., enhancing biomechanical stability or restoring functional mobility), and subsequent rehabilitation strategies are outlined.

### Workflow

The digital twin creation process followed a modular but consistent workflow across all five cases, consisting of (1) motion capture, (2) musculoskeletal simulation, (3) patient-specific image processing and segmentation, (4) virtual model generation, and (5) finite element simulation. While all cases shared this pipeline, differences arose primarily in the types of anatomical regions analyzed, the implant strategies under evaluation, and the integration of motion data. For Cases 1–4, a full-body motion capture system was used to monitor scenarios involving the lower and upper extremities under functional tasks such as walking or arm movement (see supplementary material). In Case 5, despite focusing on wrist motion, whole-body motion capture was used to ensure accurate arm segment orientation and to derive consistent kinematic constraints for the wrist joint. The setup captured segment-specific joint angles and positions, which were evaluated through an inverse dynamics pipeline within the AnyBody™ (AnyBody Technology A/S, Aalborg, Denmark) modeling system.

### Patient motion capturing

During the initial consultation and subsequent follow-up appointments throughout the patient’s recovery, a gait or motion analysis is performed under the supervision of the senior physician. The full-body motion-capturing system Xsens™ MVN Awinda (Xsens Technology B.V., Enschede, Netherlands) is used for this analysis. This system comprises 17 inertial measurement units (IMUs) placed in standardized anatomical positions across the patient’s body to capture precise segmental and whole-body motion data. The process is described in detail by Braun et al.^36^ and Andres et al.^38^. In addition to IMU-based data, specific anatomical parameters, such as segment lengths, are measured to enhance the accuracy of subsequent biomechanical simulations and analyses. The motion capture protocol was adapted to each case’s injury location and clinical objective. Standard overground walking trials were recorded for lower-extremity cases (Cases 1, 3, and 4) to simulate weight-bearing loads during gait. For the humeral fracture in Case 2, shoulder and arm movements relevant to functional loading were captured. In Case 5, where the focus was on wrist malalignment, the patient performed isolated radial and ulnar deviations under clinical supervision.

### Musculoskeletal simulation

Musculoskeletal simulations are performed using the AnyBody™ Modelling System. The AnyBody™ software system enables the generation of subject-specific biomechanical models for estimating muscle forces, joint loads, and ground reaction forces, thereby providing insights into the mechanical interactions between the musculoskeletal system and its environment. To ensure the accuracy of these simulations, key patient-specific anthropometric parameters, including height, weight, and segment lengths, are incorporated into the model configuration. The motion capture data of the patient serves as the kinematic input for the musculoskeletal simulations, providing time-resolved joint angles and segment orientations. By integrating these patient-specific parameters with recorded movement patterns, the resulting models reflect individual biomechanics, facilitating detailed assessments of musculoskeletal function under various conditions. This data then serves as boundary conditions in the biomechanical simulations, allowing a high degree of individualization^34^.

### Patient-specific image processing

Following the completion of patient-specific measurements and musculoskeletal simulations, the obtained CT scans undergo a segmentation process using the image processing software ScanIP™ (Synopsys, Mountain View, CA, United States). The anonymized digital imaging and communications in medicine (DICOM) image stacks are loaded into the software, where distinct anatomical and implant-related regions are identified based on threshold-based differences in greyscale values. Initially, automated segmentation routines are employed to create preliminary masks for bone, fracture, implant, and screws.

Subsequent manual refinements focus on areas requiring greater precision, particularly the proximal and distal bone regions and the screw trajectories. In addition, careful attention is given to accurately defining the fracture morphology and the corresponding callus area. Throughout this stage, consultation with the treating surgeon ensures a clinically appropriate segmentation and an accurate reconstruction of the patient-specific fracture configuration.

### Virtual model generation

The next step in setting up the DT involves generating a virtual representation of the patient’s treatment scenario. This reconstruction may represent a prior, current, or future surgical intervention. To achieve this, data from available CT scans, X-rays, and medical reports are thoroughly examined in conjunction with the clinical expertise of the treating surgeon. This data review ensures that the virtual model accurately reflects the anatomical and clinical realities.

A key aspect of virtual reconstruction is modifying and optimizing existing treatment strategies. For example, the model can be adjusted to change the thickness of an intramedullary nail, place or remove screws, add or remove plates, or predict wrist motion outcomes before and after a planned reconstruction surgery. Before these adjustments, the current implant situation is documented by measuring implant dimensions, assessing the type and number of screws, plate hole configurations, and intramedullary nail sizes. This inventory serves as a baseline against which planned modifications are evaluated, ensuring that the proposed changes align with clinical goals and patient-specific needs. Further optional refinements, such as integrating motion measurement data obtained before surgical revisions, can be introduced.

Upon finalizing the virtual representation, attention turns to refining the fracture and callus areas. Manual adjustments supported by detailed imaging and surgical expertise ensure that these regions accurately represent the clinical situation. In some cases, incorporating relevant boundary conditions, such as recorded motion data, may be advantageous directly into the virtual representation, enhancing the model’s predictive capacity regarding functional outcomes.

At this stage, two segmentation-based models are typically generated for each patient: one representing the current state and the other the previous or anticipated future treatment scenario. Using ScanIP™, finite element (FE) models are created for each virtual representation. Here, the image segmentation quality is first verified, after which meshing and material assignment are performed. Therefore, an adaptive meshing with quadratic tetrahedral elements of type C3D10, ten-node tetrahedra, is performed to represent the bone and implant structures. The bone material properties are derived from CT grey values following established approaches^34–36^, while the mechanical characteristics of implants, nails, and screws are chosen based on clinically relevant design parameters. The fracture properties reflect the condition of the soft callus, as described by Claes and colleagues^39,40^.

Subsequently, node sets are defined to integrate boundary and loading conditions for future simulations. Two cuboid regions at the distal and proximal ends of the bone typically serve as reference areas for applying loads or constraints. Once meshing, material assignments, and node-set configurations are complete, an input file is generated. This file contains all the necessary information to guide subsequent FE simulations.

**Fig. 8:**
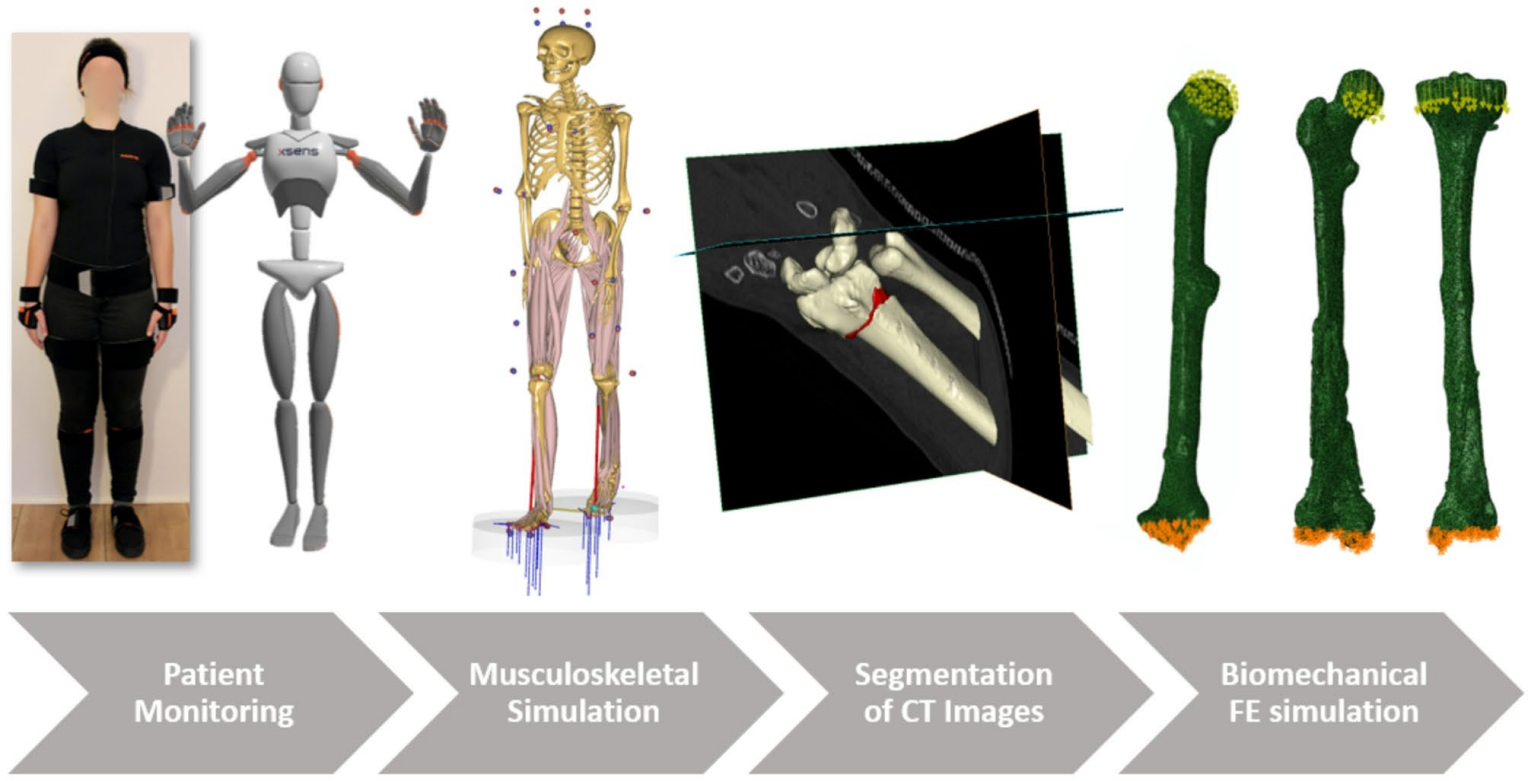
Visualization of the workflow starting with the patient monitoring, then the inverse dynamic calculation of the joint forces, the generation of the 3D models, and the FE simulation with the applied boundary conditions.

To evaluate the clinical feasibility of the proposed workflow visualized in Fig. 8, the typical time requirements for each major step: CT image processing and segmentation (8–16 h), virtual model generation (2–4 h), meshing and simulation setup (1–2 h), and finite element simulation with result post-processing (1–10 h depending on model complexity). Musculoskeletal simulations based on motion capture data require approximately 1–2 h per patient. The end-to-end process from initial imaging to mechanical analysis results can be completed within 2–3 working days. While this timeframe is incompatible with emergency settings, it aligns well with revision surgeries and complex fracture cases involving planned interventions.

### Biomechanical simulation

The input files from ScanIP™ are imported into the simulation environment Abaqus™ (Dassault Systèmes, Vélizy-Villacoublay, France). The finite element simulations were performed using the Abaqus Standard (implicit) solver and linear elastic material models. Dirichlet boundary conditions are typically applied at the bone’s distal joint side, where displacements and rotations are constrained in all three spatial axes. The applied loading conditions correspond to static representations of peak joint loads, extracted from the dynamic musculoskeletal simulations at maximum loading during representative movement (e.g., mid-stance phase in gait). While full dynamic load profiles were generated and are provided in the supplementary material, the finite element analyses used only the peak static load values to represent worst-case mechanical scenarios. Concurrently, loading conditions are imposed on the proximal bone segment, guided by musculoskeletal simulation outputs that define force vectors and load ratios. For example, as illustrated in Fig. 8, the femur is subjected to hip joint forces, while the tibia experiences knee joint forces representative of walking conditions. Similarly, relevant shoulder movements are modeled for upper arm fractures, and appropriate joint forces are applied at the shoulder.

Joint reaction forces and segmental loads were extracted from the musculoskeletal simulation at the time of maximum loading during the motion cycle, typically corresponding to mid-stance in walking or maximal muscle activation in upper-limb movement. The finite element simulations used these peak load vectors, including force directions and magnitudes, as boundary conditions. Specifically, loads were applied to the proximal joint surface of the bone models, while the distal ends were constrained. The stress and strain plots presented in the results represent the output of this peak-load simulation and reflect the condition most relevant for mechanical failure risk and decision-making.

Once the FE simulation concludes, a Python script automatically extracts essential data from the output database (ODB). This script initiates the conversion of simulation results into a visualization-friendly format using the Visualization Toolkit (VTK), capturing node labels and coordinates, element connectivity, and element sets for each mask from the segmentation and model generation steps.

Additionally, displacement vectors and stress and strain tensors are mapped to the defined masks, ensuring each structural component’s mechanical response is accurately represented. This data extraction and mapping process enables a seamless transition from raw computational outcomes to clinically more interpretable results, suitable for further analysis.

### Evaluation

To evaluate implant stability and component performance, von Mises stress distributions are analyzed, helping identify potential weaknesses in the chosen treatment configuration. However, the core focus of this study aims to understand fragment motion and how various treatments influence interfragmentary mechanics. The healing window thresholds are based on the mechanobiological criteria established by Claes et al.^39^ and Shefelbine et al.^40^. This approach involves examining octahedral shear strain, which reflects distortional deformation, and volumetric strain, indicative of volumetric changes and hydrostatic pressure in the fracture gap.

The interplay of octahedral shear and volumetric strains has been linked to cellular responses and tissue differentiation, supported by the findings of Bishop and colleagues^41^, Garcia and colleagues^42^, and Doblaré and colleagues^43^. Each tetrahedral element in the FE model is individually analyzed to determine the local strain tensor. Subsequently, the octahedral shear and volumetric strains are calculated and evaluated within the specified limits established by the adopted mechanobiological modeling approach^39,40^. This analysis offers patient-specific and treatment-related insights into the mechanical environment surrounding the fracture, informing treatment strategies and guiding more effective, personalized orthopedic interventions.

## Electronic supplementary material

Below is the link to the electronic supplementary material.

Supplementary Material 1

## Data Availability

The original contributions presented in the study are included in the article; further inquiries can be directed to the corresponding author. Researchers who wish to request access to data should send an email indicating the research purpose. Every request must be reviewed by the responsible institutional review boards, considering the risk of patient reidentification and compliance with the applicable data protection rules.
