# Supplementary material for: Advantages of digital twin technology in orthopedic trauma Surgery – Exploring different clinical use cases
Source: Sci Rep. 2025 Jun 6;15:19987. doi: 10.1038/s41598-025-04792-w (PMC12144170; doi:10.1038/s41598-025-04792-w)
Supplement: Supplementary file 1 — Supplementary Material 1 [file 41598_2025_4792_MOESM1_ESM.docx]

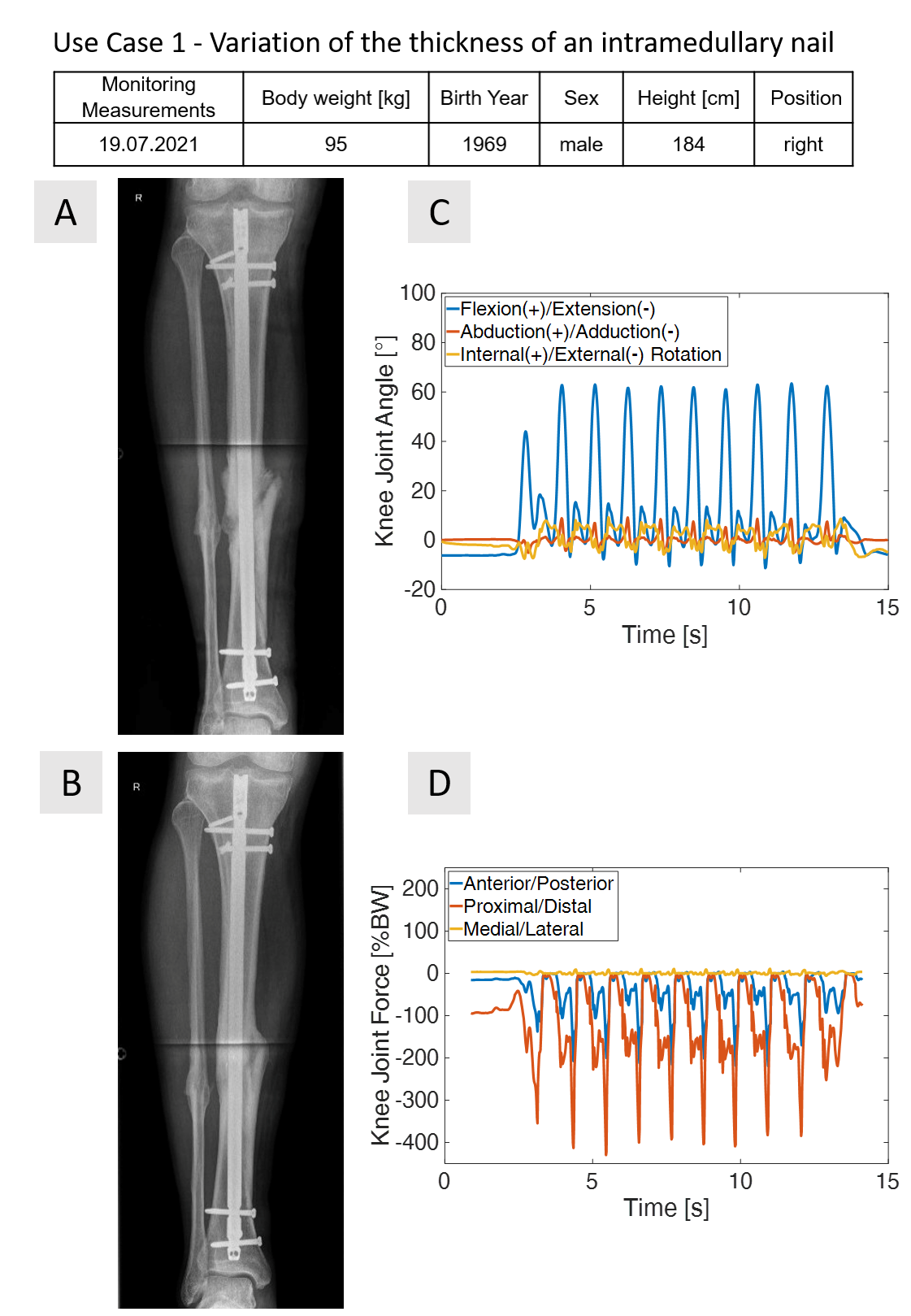


Figure 1:Supplementary material for the use case 1 with additional patient information and A) preoperative X-ray, B) postoperative X-ray, C) motion capturing data, and D) joint force data.


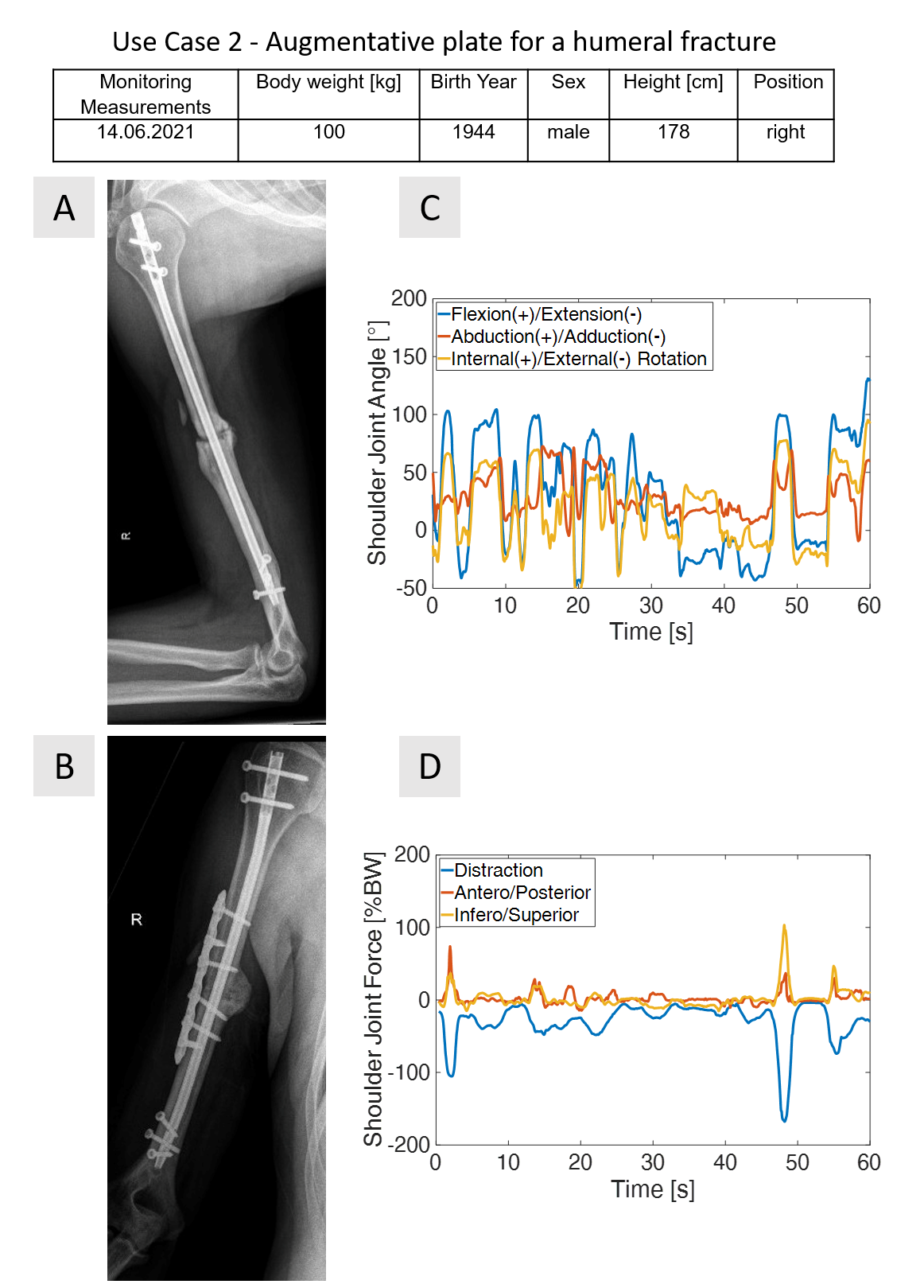


Figure 2: Supplementary material for the use case 2 with additional patient information and A) preoperative X-ray, B) postoperative X-ray, C) motion capturing data, and D) joint force data.


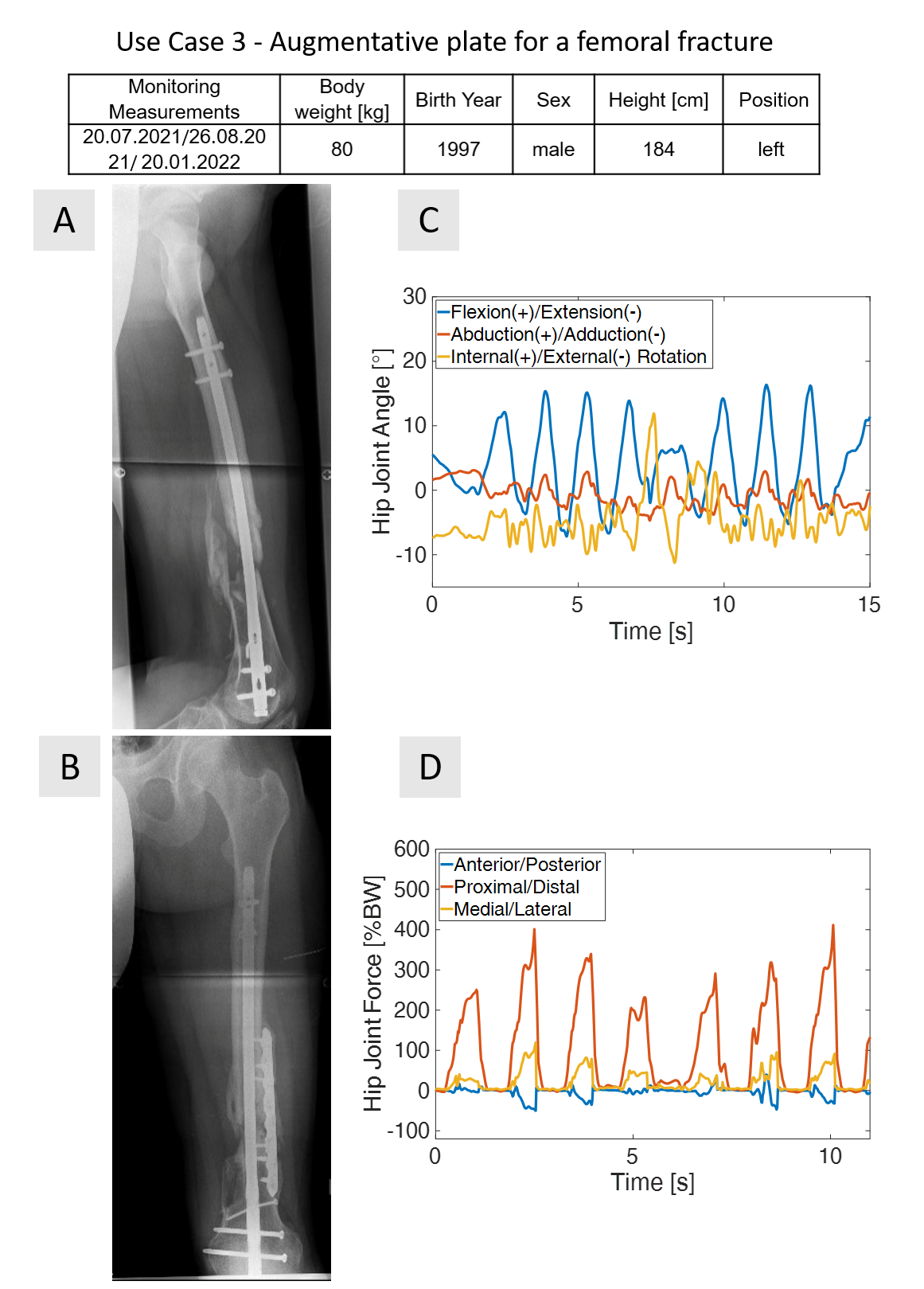


Figure 3: Supplementary material for the use case 3 with additional patient information and A) preoperative X-ray, B) postoperative X-ray, C) motion capturing data, and D) joint force data.


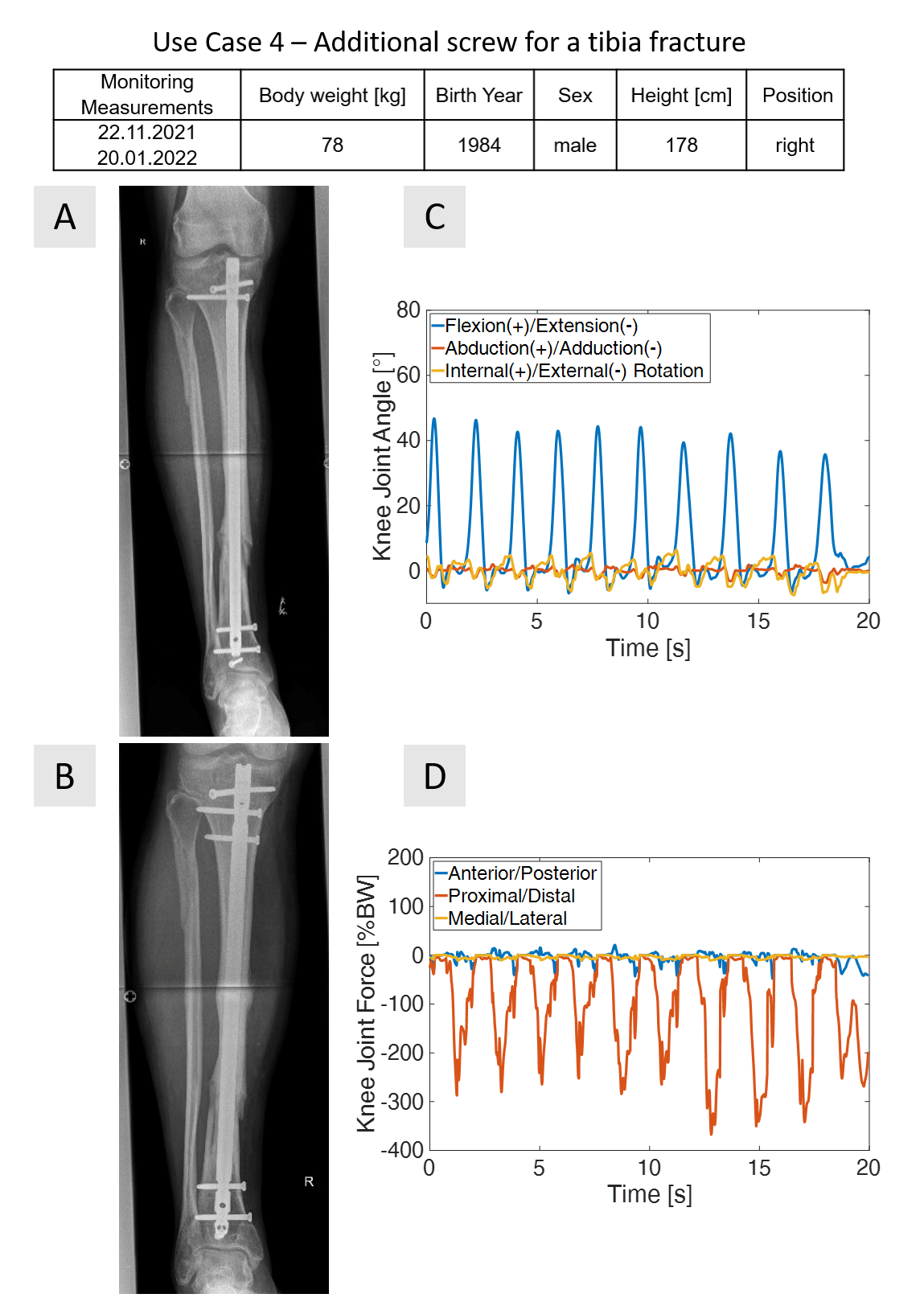


Figure 4: Supplementary material for the use case 4 with additional patient information and A) preoperative X-ray, B) postoperative X-ray, C) motion capturing data, and D) joint force data.


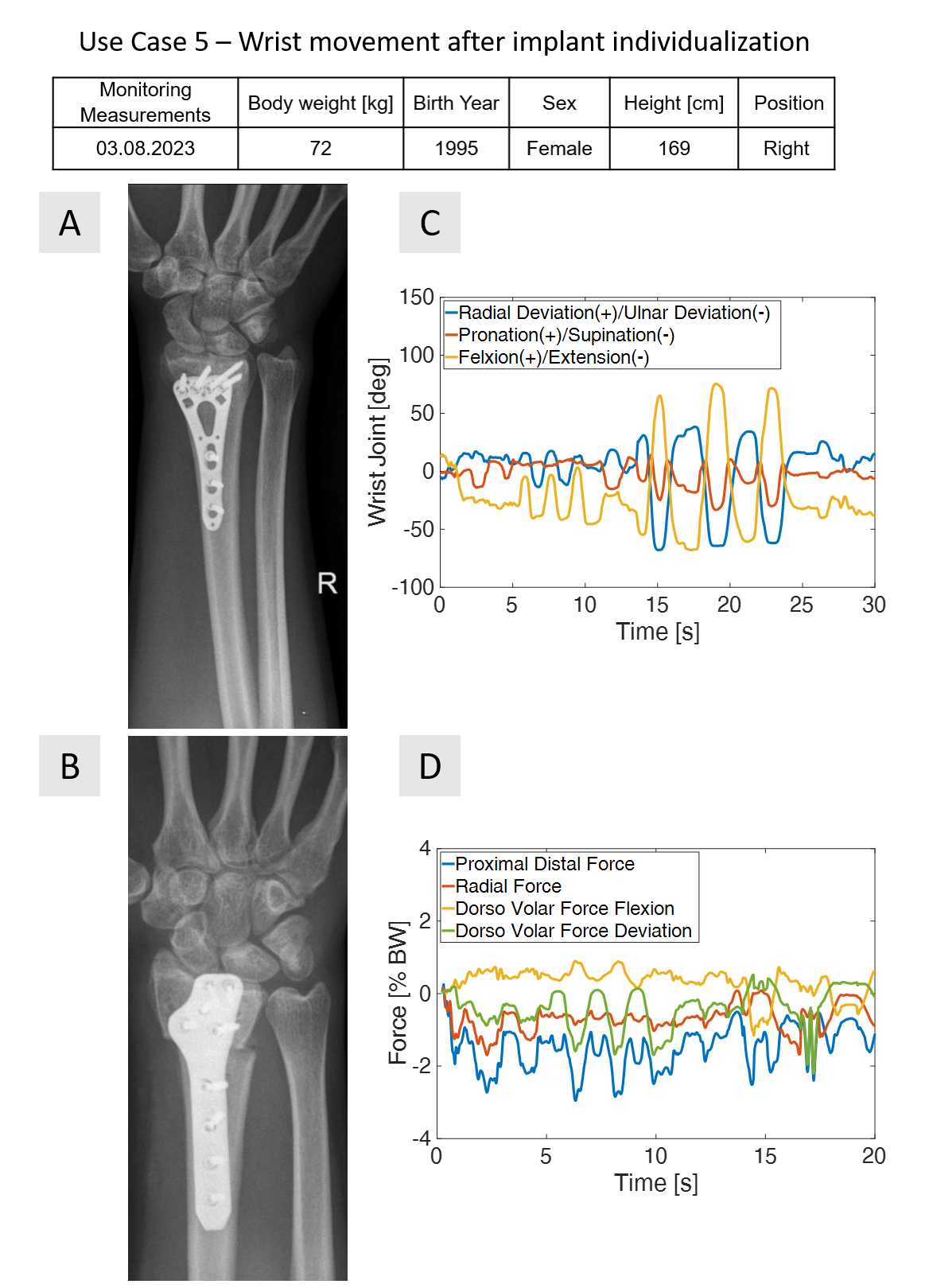


Figure 5: Supplementary material for the use case 5 with additional patient information and A) preoperative X-ray, B) postoperative X-ray, C) motion capturing data, and D) joint force data.


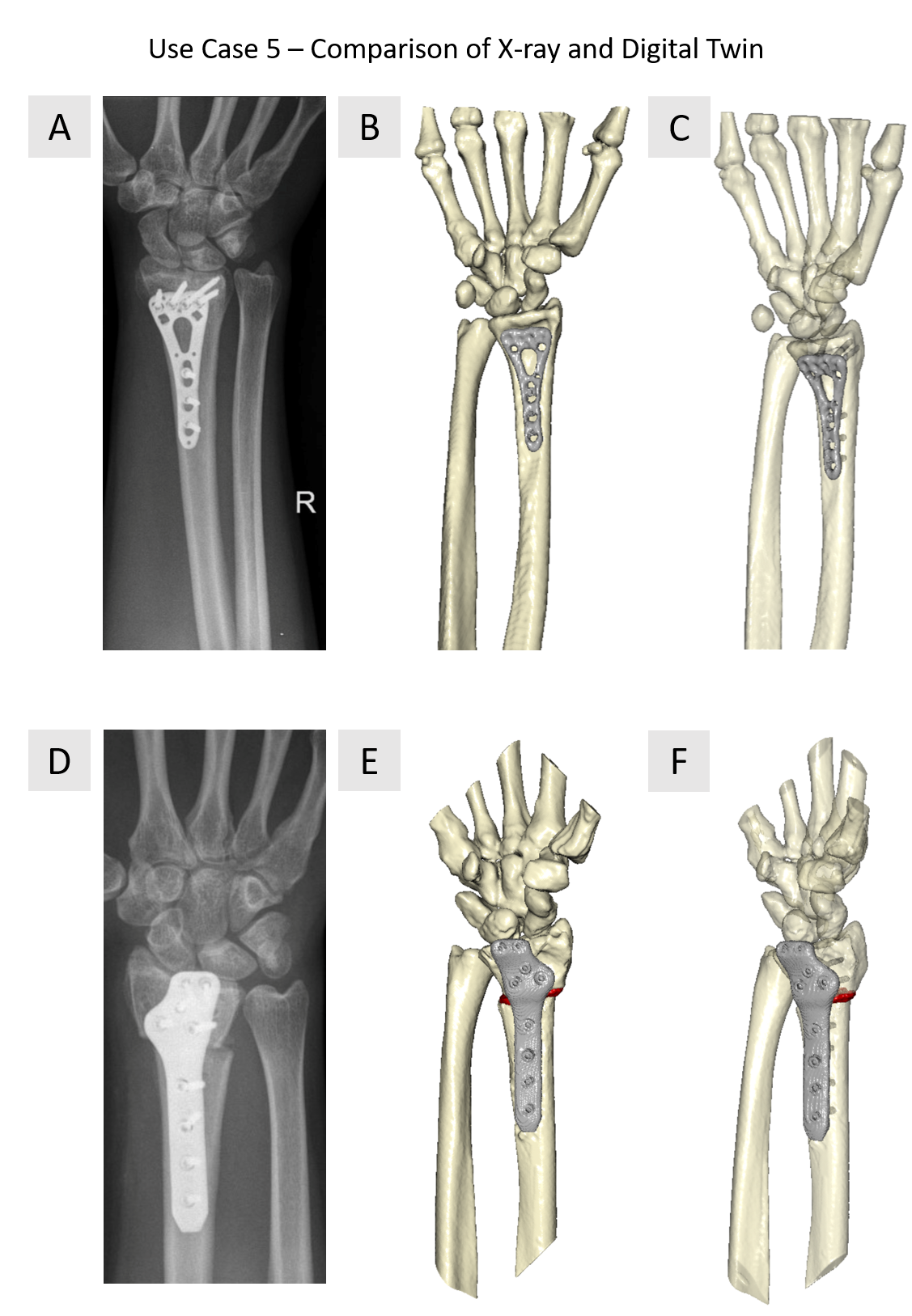


Figure 6: Example comparison of X-ray images and digital twin for the use case 5. A) shows the X-ray for the initial treatment, B) the digital twin with an opacity of 1, and C) the digital twin with an opacity of 0.4 for the initial treatment. D) shows the X-ray for the current treatment, E) the digital twin with an opacity of 1, and F) the digital twin with an opacity of 0.4 for the current treatment.

| **Use Case** | **Maximum von Mises stress of the implant in the fracture area [Mpa]** | | **Healing window coverage [%]** | |
| --- | --- | --- | --- | --- |
| **Treatment** | **Initial** | **Current** | **Initial** | **Current** |
| 1 | 120 | 58 | 4.5 | 0 |
|  |  |  | 2.5 | 0 |
|  |  |  | 74.7 | 72 |
|  |  |  | 18.3 | 28 |
|  |  |  | 0 | 0 |
| 2 | 240 | 78 | 5.6 | 0 |
|  |  |  | 3.3 | 0 |
|  |  |  | 91.1 | 97.8 |
|  |  |  | 0 | 2.2 |
|  |  |  | 0 | 0 |
| 3 | 160 | 30 | 1.2 | 0 |
|  |  |  | 85.5 | 0 |
|  |  |  | 1.1 | 74.6 |
|  |  |  | 9 | 14.9 |
|  |  |  | 0.2 | 10.5 |
| 4 | 53 | 57 | 0 | 0 |
|  |  |  | 0 | 0 |
|  |  |  | 76 | 86 |
|  |  |  | 21.6 | 14 |
|  |  |  | 2.4 | 0 |
|  | | | | |
| **Use Case** | **Contact** | |  | |
| **Treatment** | **Initial** | **Current** |  |  |
| 5 | Contact triquetrium and ulna | No contact |  |  |

Figure Legends

Figure 1: Supplementary material for the use case 1 with additional patient information and A) preoperative X-ray, B) postoperative X-ray, C) motion capturing data, and D) joint force data.

Figure 2: Supplementary material for the use case 2 with additional patient information and A) preoperative X-ray, B) postoperative X-ray, C) motion capturing data, and D) joint force data.

Figure 3: Supplementary material for the use case 3 with additional patient information and A) preoperative X-ray, B) postoperative X-ray, C) motion capturing data, and D) joint force data.

Figure 4: Supplementary material for the use case 4 with additional patient information and A) preoperative X-ray, B) postoperative X-ray, C) motion capturing data, and D) joint force data.

Figure 5: Supplementary material for the use case 5 with additional patient information and A) preoperative X-ray, B) postoperative X-ray, C) motion capturing data, and D) joint force data.

Figure 6: Example comparison of X-ray images and digital twin for the use case 5. A) shows the X-ray for the initial treatment, B) the digital twin with an opacity of 1, and C) the digital twin with an opacity of 0.4 for the initial treatment. D) shows the X-ray for the current treatment, E) the digital twin with an opacity of 1, and F) the digital twin with an opacity of 0.4 for the current treatment.

Table Legend

Table 1: Summary table across the five cases.
